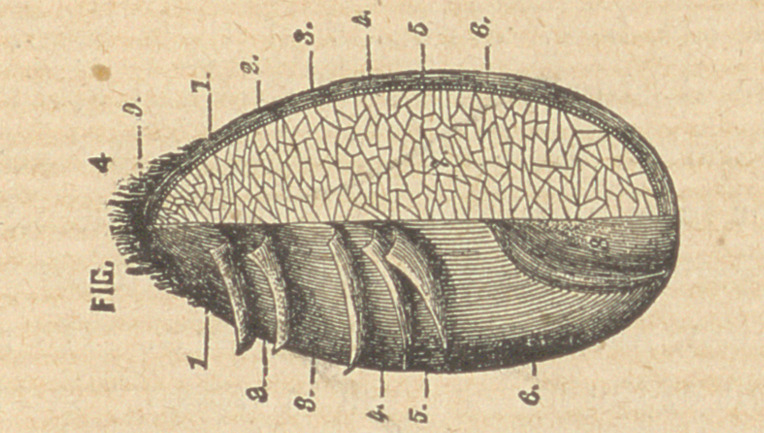# The Curse of Modern Civilization

**Published:** 1883-10

**Authors:** George C. Brown

**Affiliations:** Elizabeth, New Jersey


### THE CURSE OF MODERN CIVILIZATION.

BY DR. GEORGE C. BROWN, ELIZABETH, NEW JERSEY.

I have a little matter to call your attention to, which I think is one of the most vitally important questions of the day, not only for the D. D. S. and the M. D. to consider, but for every intelligent, thinking human being, and because it is not so considered is the reason I have called it the Curse of Modern Civilization—white flour. My object in writing this paper is to call your attention to the minute anatomy of a grain of wheat, and by this examination to gain, if possible, a little light on the subject of phosphate food. If I can in this way get you to thinking on the subject, and then we all by a combined effort be enabled to start the reform ball rolling, I shall think that we are on the true road to perfection, the preventive rather than the curative, and who knows but that it may be the destiny of the dentist by his researches and conferences to finally point out to suffering humanity the true elixir of life—health without physic.

Let us look at the construction of a grain of wheat.

Fig. 1 represents a grain of wheat in its natural state, with the husk and fibrous beard on. This husk is the only indigestible and innutritious part of the grain, being composed of silex and woody fibre; if this be carefully removed you will find the grain as represented in fig. 2.

Fig. 3 represents a section of the grain highly magnified,

1, 1, 2, 3, 4, constitute what is commonly known as bran; 1, 1, 2, being the husk, and 3, 4, 5, 6, containing the food elements, which can be more easily seen in fig. 4.

Nos. 1, 2, 3, 9, constitute the outer husk and fibrous beard.

Nos. 4, 5, 6, 8, constitute that portion of the wheat wherein reside nearly all the mineral elements.

No. 7 represents the starch cells and constitutes about seven-tenths of the entire berry.

The starch has no properties capable of building up the system and repairing its vital waste; its only action is as fuel, and with fat it supplies the heat which is necessary to keep up the steam. To carry out the illustration, Nos. 4, 5, 6, 8, supply the water for the steam engine, from which the steam (vital force) is formed, and you are all aware of the effects of a hot fire under a boiler with no water in it. This is just the condition when only fine flour is used. The entire outside of the berry down to the starch is removed, and they send us hell fire in the beautiful disguise of white flour.

An analysis of wheat and white flour gives the following result from 1000 parts of substance:

| | | |
|---|---|---|
| Wheat has an ash of...... | 17.7 | parts. |
| White Flour has an ash of | 4.1 | parts—an impoverishment of about $\frac{3}{4}$. |
| Wheat has................ | 8.2 | parts phosphoric acid. |
| White Flour has.......... | 2.1 | parts phosphoric acid—an impoverishment of about $\frac{3}{4}$. |
| Wheat has ............... | 0.6 | parts lime and 0.6 parts soda. |
| White Flour has.......... | 0.1 | parts lime and 0.1 parts soda—an impoverishment of 5-6 in lime and soda each. |
| Wheat has.... .......... | 1.5 | parts sulphur—White Flour has no sulphur. |
| Wheat has............... | 0.5 | parts sulphuric acid—White Flour has no sulphuric acid. |

A Boston physician, in a paper read before the New Hampshire Medical Society, made the following statement:

"I became alarmed at the decay of my children's teeth—I queried dentists and others as to the cause, and settled down to the conclusion that there was something wrong in the bony elements of their food. Subsequently Dr. Nichols, editor of the Boston Journal of Chemistry, brought to my notice some comparative chemical analyses of wheat and flour, showing the great deficiency in the latter in mineral ash. I was then using in my family a celebrated brand of flour called 'Peerless.' I took this flour to the State Assayer, Prof. Sharples of Boston, and had him analyze it. He reported withdrawal of seventy-five per cent of the mineral ash in wheat." And he further says, "I still think that, had I not had this analysis made and paid for it, I should not have been so thoroughly convinced as I now am, of the terrible impoverishment in flour."

Majendie in his experiments on this subject found that dogs fed on white flour died in about forty days, of starvation, while dogs fed on flour made from the entire wheat throve and grew strong.

The question now is what shall we do for a substitute, as I think I have proven to you that white flour is not a proper food for human beings, and as I do not think it necessary to call your attention to what you all see every day in your offices—that is the lack of the phosphates and nitrates in our patients, especially the children, —I take it for granted that you all feel the want.

Let us look for a substitute.

Oat meal has been a great help to us, but you will all agree that it does not come anywhere near what is required.

Graham flour has been used and considered a good article, but if we look at the way it is made we will have to discard this too.

There are two improper ways of preparing brown flour; the first is by grinding the wheat whole, so that the indigestible and irritating husk (which is not only unhealthy in itself but contains the eggs and larvæ of insects) forms part of the flour, and is certainly injurious; the other way is by mixing poor white flour with bran, and the result is the same.

Meat and vegetables contain the same elements to a certain extent, but there is no other food that combines them like wheat, in nearly the exact proportion in which they are contained in the human system, so that it is evident that wheat was intended for the principal food of man and the only way to get its whole virtue is to remove the outer husk and grind all the rest; this will produce a dark flour, varying in color with the soil in which is grown the wheat from which it was made.

## Figures and Tables

**Figure f1:**
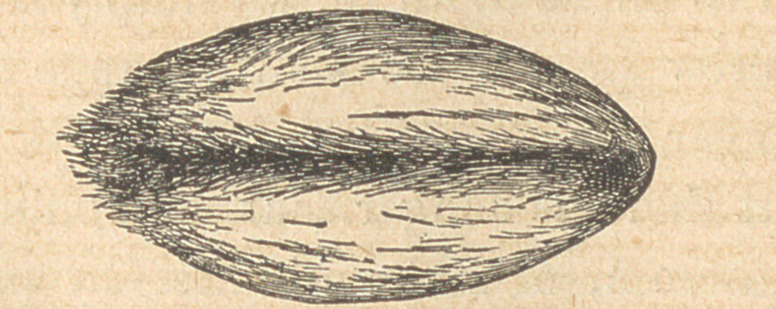


**Figure f2:**
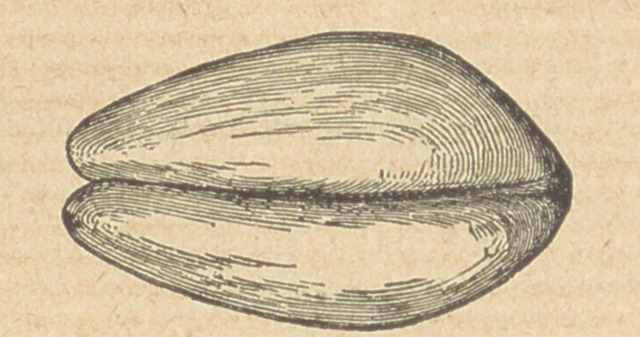


**FIG. 3 f3:**
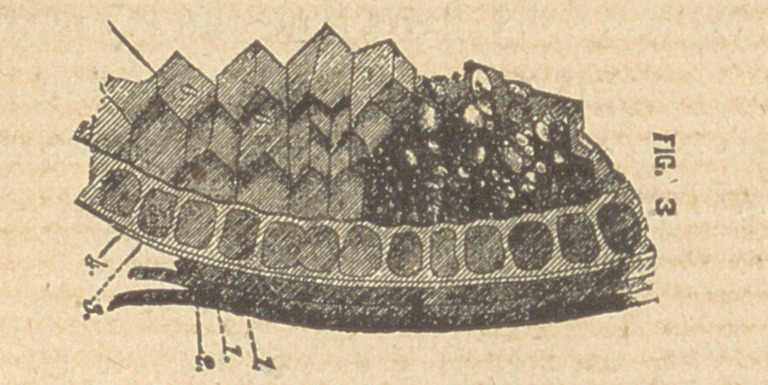


**FIG. 4 f4:**